# Study protocol for comparing the efficacy of left-open single-flap technique versus double-flap technique after proximal gastrectomy: A multicenter randomized controlled trial

**DOI:** 10.3389/fonc.2022.973810

**Published:** 2022-11-17

**Authors:** Qin Chuan Yang, Wei Dong Wang, Zhen Chang Mo, Chao Yue, Hai Kun Zhou, Rui Qi Gao, Juan Yu, Dan Hong Dong, Jin Qiang Liu, Jiang Peng Wei, Xi Sheng Yang, Gang Ji, Xiao Hua Li

**Affiliations:** ^1^ Department of Gastrointestinal Surgery, Xijing Hospital, Air Force Military Medical University, Xi’an, Shaanxi, China; ^2^ Shaanxi University of Chinese Medicine, Xi’an, Shaanxi, China

**Keywords:** gastric cancer, double-flap technique, left-open single-flap technique, proximal gastrectomy, study protocol

## Abstract

**Background:**

Proximal gastrectomy has gradually gained more attention due to its superiority in retaining the function of part of the stomach. The inevitable loss of the antireflux barrier and postoperative complications resulting from proximal gastrectomy can severely affect the quality of life. Continuous improvements in digestive tract reconstruction after proximal gastrectomy have yielded the development of a variety of methods with antireflux functions. Recently, our center attempted the left-open single-flap technique and initiated a multicenter, prospective, randomized controlled trial for patients undergoing proximal gastrectomy to reduce the difficulty of surgical anastomosis and the incidence of perioperative complications compared with the double-flap technique. These findings will provide more evidence-based medical research for the development of clinical guidelines.

**Methods/design:**

This study is a prospective, multicenter, randomized controlled clinical trial. We plan to recruit 250 patients who are eligible for proximal gastrectomy. After informed consent is obtained, patients will be randomly assigned to the trial group (left-open single-flap technique) and the control group (double-flap technique) in a 1:1 allocation ratio.

**Discussion:**

Increasingly, clinical studies have focused on the improvement of reconstruction modalities after proximal gastrectomy. Among these methods, the double-flap technique is a clinically effective method. The purpose of this study is to establish a prospective randomized controlled trial to compare the efficacy of the left-open single-flap technique versus the double-flap technique after proximal gastrectomy, aiming to provide more evidence-based medical studies for digestive tract reconstruction in proximal gastrectomy.

**Clinical Trial Registration:**

ClinicalTrials.gov, identifier [NCT05418920].

## Introduction

Despite the decreasing incidence and mortality of gastric cancer in recent years, the incidence and proportion of proximal early gastric cancer has markedly increased (1). However, the choice of an appropriate surgical procedure, the only radical treatment for this condition, has been controversial (2). Currently, proximal gastrectomy has gradually gained increasing attention due to its superiority in 1) maintaining the distal gastric volume; 2) preserving the fundic gland area and reducing hormonal and nutritional deficiencies; and 3) ensuring the secretion of internal factors and gastric acid, the absorption of iron ions and vitamin B12, and the maintenance of hemoglobin concentration (3–6). However, the inevitable a loss of the antireflux barrier and postoperative complications resulting from proximal gastrectomy can severely affect the quality of life (7).

Recently, continuous improvements in digestive tract reconstruction after proximal gastrectomy have yielded the development of a variety of methods with antireflux functions that contribute to retaining the function of the residual stomach and avoiding serious reflux esophagitis (8–10). Japanese guidelines indicate that popular methods for gastrointestinal reconstruction after proximal gastrectomy include esophagogastrotomy (EG), jejunal interposition (JI), and double-tract reconstruction (DTR) (11–13). In 2016, Kuroda reported a double-flap technique using the anterior gastric wall plasma muscle flap to cover the anastomosis (14). Its unidirectional flap can reconstruct the “sphincter” and reduce the incidence of reflux esophagitis and the risk of anastomotic fistula.

By comparing patients who underwent direct esophagogastrostomy, jejunal interposition, double tract reconstruction, and the double-flap technique, Shoji Y (15) and Saze Z et al. (16) found that patients undergoing double-flap anastomosis had no reflux esophagitis and a lower incidence of postoperative anastomotic stricture, which improved postoperative serum albumin ratio changes and weight maintenance. Consequently, the double-flap technique is considered to be the most effective technique for proximal gastric reconstruction (17, 18).

However, the double-flap technique is associated with shortcomings, such as a more complex suture technique, more difficult operation, longer operation time and higher incidence of postoperative anastomotic stenosis (15, 16, 19). Therefore, our center attempted the left-open single-flap technique to reduce the difficulty of surgical suturing and the incidence of complications. To further evaluate the therapeutic efficacy of this procedure, we initiated a multicenter, prospective, randomized controlled trial for patients undergoing proximal gastrectomy, which will provide more evidence-based medical research for the development of clinical guidelines.

## Trial objectives

This study is a prospective, multicenter, randomized controlled clinical trial. We plan to recruit 250 patients who are eligible for proximal gastrectomy. After informed consent is obtained, patients will be randomly assigned to the trial group (left-open single-flap technique) and the control group (double-flap technique) in a 1:1 allocation ratio, with the aim of providing more evidence-based medical outcomes for digestive tract reconstruction in proximal gastrectomy. The surgical methods applied in this study are shown in **Table 1**.

**Table 1:** Surgical methods applied in this study.

	Proximal gastrectomy	D2 lymphadenectomy	Left-open single-flap technique	Double-flap technique
The trial group	√	√	√	
The control group	√	√		√

The objectives of this trial are as follows:

### Main objective

The main objective is to investigate the incidence of reflux esophagitis after 2 types of anastomosis (left-open single-flap technique vs. double-flap technique) after proximal gastrectomy.

### Secondary objective

1) to investigate the incidence of anastomotic leakage of 2 types of anastomosis (left-open single-flap technique vs. double-flap technique) after proximal gastrectomy
2) to investigate the incidence of anastomotic stricture of 2 types of anastomosis (left-open single-flap technique vs. double-flap technique) after proximal gastrectomy
3) to investigate the operation time of 2 types of anastomosis (left-open single-flap technique vs. double-flap technique) after proximal gastrectomy
4) to investigate the intraoperative blood loss volume of 2 types of anastomosis (left-open single-flap technique vs. double-flap technique) after proximal gastrectomy

## Prospective results

In patients after proximal gastrectomy, the left-open single-flap technique can decrease postoperative complications and increase nutritional status compared with the double-flap technique.

## Participant selection

**Figure 1:**
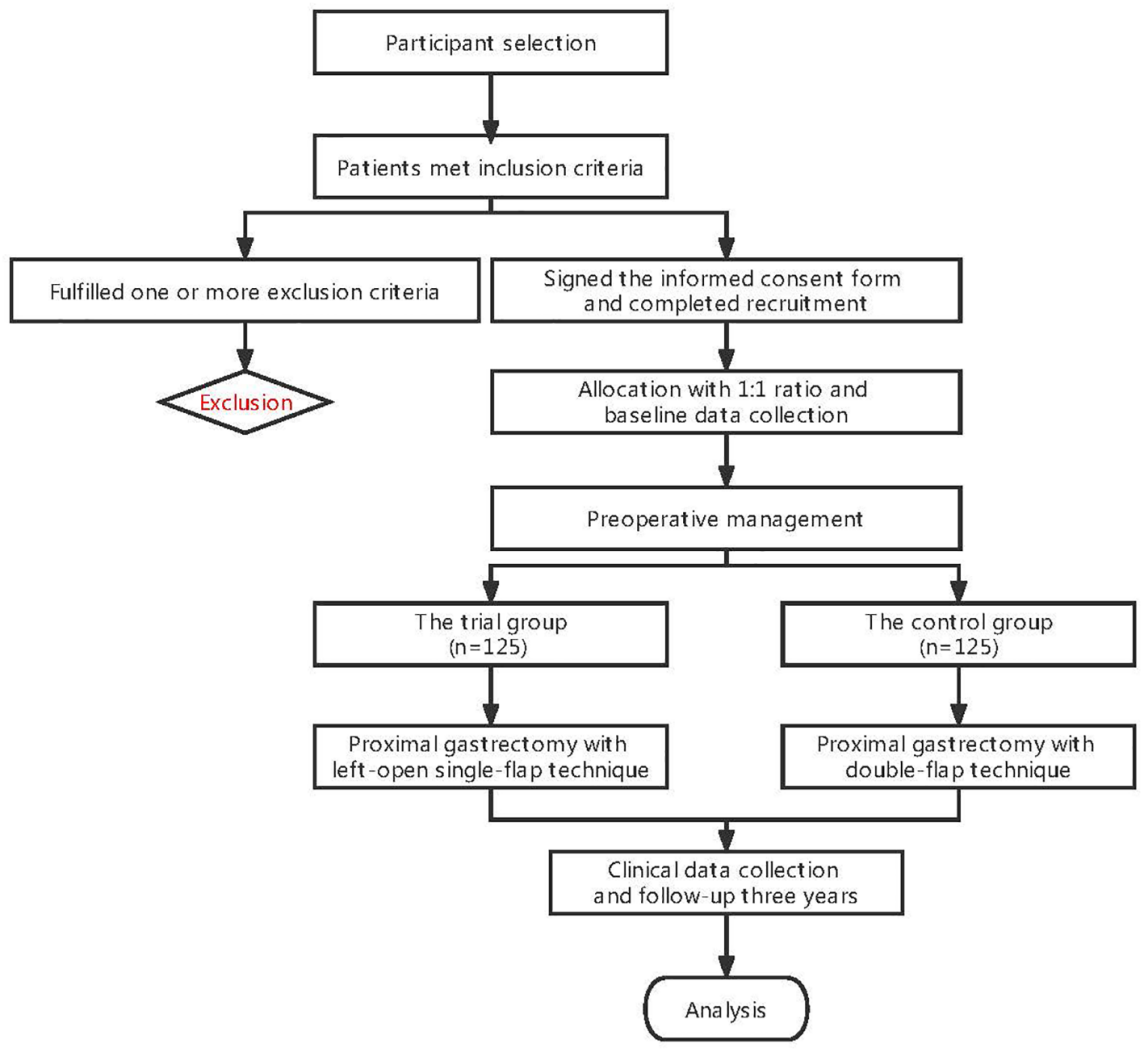
Flowchart of the trial.

The flow chart of this study is shown in **Figure 1**. During the routine admission of inpatients, suitable patients will be screened by the study staff according to the inclusion/exclusion criteria. Patients who are successfully selected before formal enrollment will receive study instructions from the investigator with a detailed explanation of the included documents and operations. Participants (or their legally authorized representative) will agree to sign and date the informed consent form after receiving a random serial number. All processes will strictly follow the provisions of the Ethical Review of Biomedical Research Involving Humans (Trial), the Declaration of Helsinki v.08, and the International Ethical Guidelines for Biomedical Research Involving Humans.

### Inclusion criteria

1) patients aged 18-80 years, regardless of sex;
2) Siewert III of esophagogastric junction adenocarcinoma: Stage I (cT_1-2_N_0_M_0_) or adenocarcinoma of the upper part of the stomach: Stage II (cT_1-2_N_0_M_0_), Stage II (cT_1-2_N_1-3_M_0_/cT_3-4_N_0_M_0_), Stage III (cT_3-4a_N_1-3_M_0_). All patients were selected according to the 8th AJCC clinical staging of gastric cancer.
3) primary lesion diagnosed by preoperative endoscopic end pathology: tumor diameter <4 cm and located in the upper part of the stomach (including the esophagogastric junction), histologically confirmed adenocarcinoma;
4) preoperative ASA score: I, II, or III;
5) preoperative Karnofsky physical status score: ≥ 70%; or preoperative ECOG physical status score: ≤ 2;
6) no distant metastases (confirmed by preoperative chest X-ray, abdominal ultrasound, and upper abdominal CT); No peritoneal implant metastases (confirmed by exploration surgery);
7) R0 surgical outcome was expected to be obtained with D2 lymphadenectomy in radical proximal gastrectomy;
8) patients and their families voluntarily participated in this study and signed the informed consent form after understanding the study content.

### Exclusion criteria

1) patients who have received any preoperative treatment, such as chemotherapy, radiotherapy, targeted therapy or immunotherapy; preoperative neoadjuvant chemotherapy recipients;
2) patients with clinical stage exceeding Siewert III of the esophagogastric junction adenocarcinoma: Stage I (cT_1-2_N_0_M_0_) or more than adenocarcinoma of the upper part of the stomach: Stage I (cT_1-2_N_0_M_0_), Stage II (cT_1-2_N_1-3_M_0_/cT_3-4_N_0_M_0_), Stage III (cT_3-4a_N_1-3_M_0_);
3) patients with acute infections, especially biliary tract infections;
4) patients with complications of gastric cancer (bleeding, perforation, or obstruction) requiring emergency surgery;
5) patients with uncorrectable coagulation dysfunction;
6) patients with vital organ failure, such as heart, lung, liver, brain, kidney, etc.
7) severe central nervous system disease, mental disorders, or impaired consciousness;
8) pregnant or lactating women;
9) patients with distant metastases;
10) patients with a primary tumor at another site diagnosed within the past 5 years;
11) preoperative ASA score: ≥ IV;
12) preoperative ECOG physical status score: ≥ 2;
13) history of continuous systemic corticosteroid therapy within the past 1 month;
14) history of unstable angina, myocardial infarction, cerebral infarction, or cerebral hemorrhage within the past 6 months;
15) patients with concurrent surgical treatment of other diseases;
16) patients with immunodeficiency, immunosuppression, or autoimmune diseases (organ transplant requiring immunosuppressive therapy within the past 5 years, allogeneic bone marrow transplant patients, taking immunosuppressive drugs, etc.);
17) patients with concurrent participation in other clinical studies;
18) patients refusing to sign an informed consent form to participate in this study;
19) preoperative imaging: regional fusion of enlarged lymph nodes (maximal diameter > 3 cm).

### Terminating criteria

1) patients are inoperable for various reasons after recruitment;
2) the investigator considers that the patient should stop this study for safety reasons or the benefit of the patient;
3) serious complications or intolerable adverse reactions of the patient;
4) patients may request to withdraw/terminate from the trial at any time after signing the informed consent form.

### Rejecting criteria

1) patients with missing main observation indicators and significantly incomplete study data;
2) incomplete follow-up data;
3) patients who failed to follow the study protocol;
4) the study protocol was discontinued after the patient was judged to be a culled case. Follow-up treatment was determined by the investigator according to clinical guidelines. The excluded cases were still subject to follow-up and were included in the study analysis.

## Participating entities

As shown in **Table 2**, this work is a multicenter, large-sample clinical study with six participating medical institutions (Xi-Jing Hospital, Tang-Du Hospital, First Affiliated Hospital Xi’an Jiaotong University, General Hospital of Ningxia Medical University, Henan Provincial People’s Hospital, The First Affiliated Hospital of Shanxi Medical University). The mode of enrollment is competitive. All study institutions and personnel were approved by the ethics committee and possessed extensive clinical experience in the treatment of gastric cancer.

**Table 2:** The perioperative follow-up data were sorted and merged.

Number	Center	Role
1	Xi-Jing hospital	Management
2	Tang-Du Hospital	Participant
3	First Affiliated Hospital Xi'an Jiaotong University	Participant
4	General Hospital of Ningxia Medical University	Participant
5	Henan Provincial People's Hospital	Participant
6	The First Affiliated Hospital of Shanxi Medical University	Participant

## Blinding technique and randomization procedure

The study has an open design.

Before randomization, the oncologic evaluation will be performed based on relevant clinical parameters (vital signs, serum biochemical tests, tumor markers, CT and/or MRI, ultrasound endoscopy, etc.). Eligible participants will be informed by the investigator and required to sign an informed consent form. Patients will be randomly assigned in a 1:1 ratio to the trial group (left-open single-flap technique) and the control group (double-flap technique). The randomization sequence will be generated by a biostatistician using the SPSS 28.0.1 software. The randomization list will be sealed in an opaque envelope and placed in the custody of a dedicated person.

None of the assistants associated with the randomization process are directly involved in this study to avoid bias.

## Perioperative management

1) If the patient’s condition deteriorates between enrollment and the date of surgery, the investigator will decide whether to perform the surgery as planned. If emergency surgery or cancellation is needed, the case will be excluded according to the exclusion criteria.
2) Perioperative enteral/parenteral nutrition support will be allowed for patients with nutritional risk.
3) For high-risk patients (elderly patients, smokers, diabetic patients, obese patients, or patients with a history of chronic cardiovascular or thromboembolic disease), the perioperative administration of low-molecular heparin, lower extremity antithrombotic compression stockings, aggressive lower extremity massage and respiratory function training are recommended as prophylactic measures. Methods for other potentially high-risk complications will be determined by clinical practice routines and specific needs, but all measures need to be documented in the CRF.
4) Regarding the choice of surgical procedure performed in this study, D2 lymphadenectomy in radical proximal gastrectomy will be performed by the investigator according to the 6th edition of the Japanese Guidelines for the Treatment of Gastric Cancer (18);
5) The principles of prophylactic antibiotic use are as follows: the first intravenous drip is started 30 minutes before surgery, and the recommended choice is cephalosporin II antibiotics. The preparation, concentration, and infusion rate will be in accordance with routine clinical methods. Prophylactic use will last no longer than 3 days after surgery, and the frequency of use will be 1 time/8 hours. In cases of allergy to cephalosporins (including a history of allergy or allergy after use), other types of antibiotics will be selected according to clinical specifics, and the duration of prophylactic use will be the same as before.
6) The patient’s preoperative fasting, water fasting, and other anesthetic requirements will be implemented according to the routine anesthetic protocol;
7) The investigator will decide to leave a gastric tube or drainage tube in place based on experience and actual needs;
8) Enhanced Recovery After Surgery program: treat preoperatively anemia with intravenous or iron to diminish blood transfusion; malnutrition treatment with hyperproteic nutritional shakes; aerobic exercise daily of 30-45 minutes; anxiety treatment with mindfulness exercise and/or drugs.

## Surgical principles

1. Rules for gastric resection:
2. Routine abdominal exploration will be performed to confirm the presence of peritoneal implants, positive abdominal exfoliative cytology, or other distant metastases and to identify those who cannot be resected due to tumor;
3. Proximal gastrectomy should be performed if the tumor is confirmed to be radical;
4. For patients who require total gastrectomy or combined organ resection intraoperatively, whether to proceed to laparoscopic surgery or intermediate open surgery is decided on a case-by-case basis. These cases are not required in this study and need to be recorded in the CRF.
5. Rules for lymph node dissection: D2 lymphadenectomy in radical proximal gastrectomy will be performed according to the tumor infiltration (17, 18).

## Treatment protocols and surgical intervention

The mesenterium at the root of the esophagus is fully stripped, and the esophagus is exposed. The location of the tumor is localized according to intraoperative gastroscopy, and the sites of esophageal and gastric body dissection are further clarified (the distances between the cut edges are 5 cm and 3 cm, respectively). The esophagus is dissected, and the stomach is pulled outside the body through an adjuvant incision in the navel or epigastrium. The tumor and remnant stomach are then dissected with linear staplers. Specimens are taken at the cut edge for rapid cytopathological examination.

**Figure 2:**
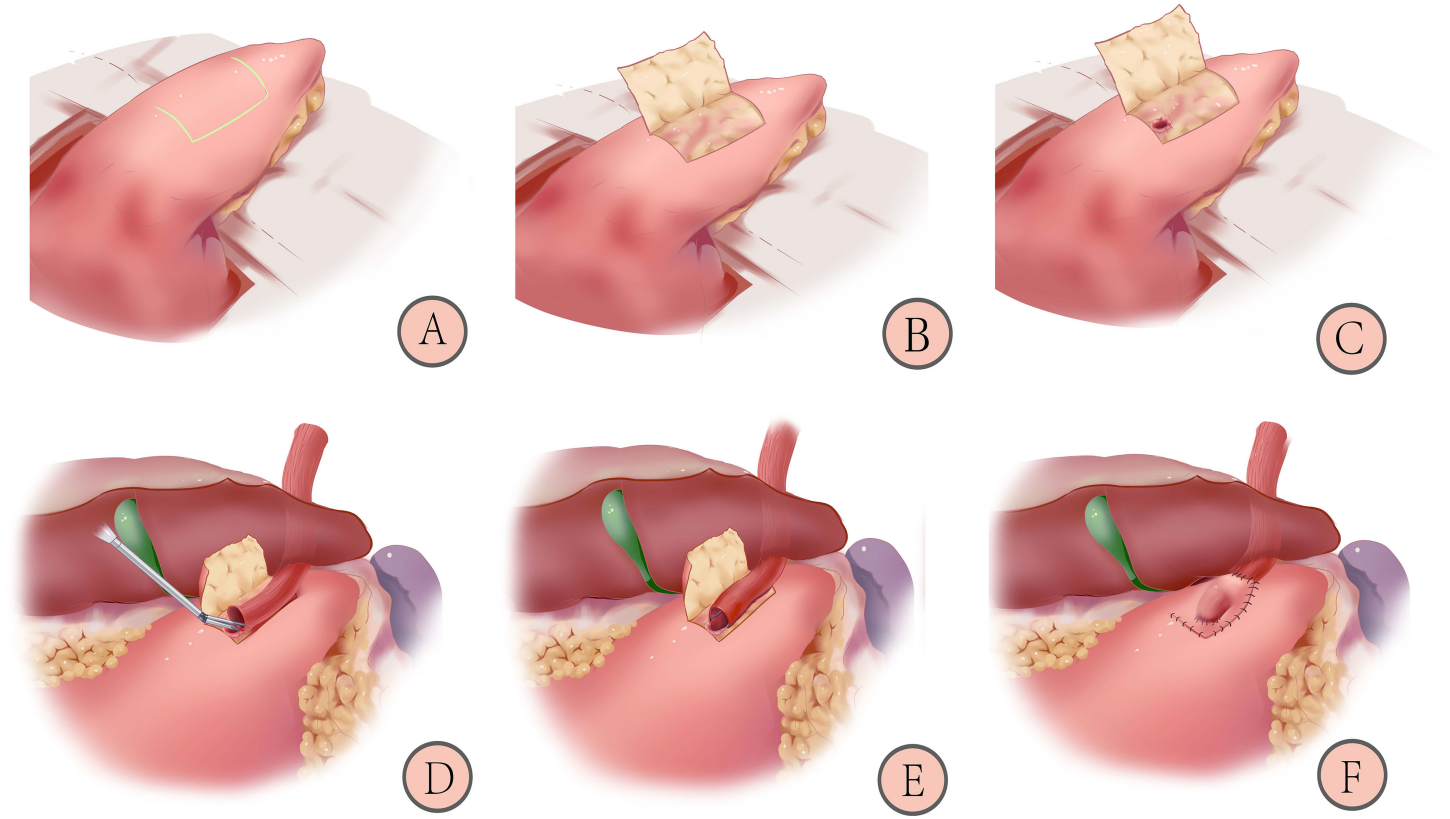
Schematic diagram of reconstruction after proximal gastrectomy with left-open single-flap technique: 1) Marking the “⊐”-shaped mucosal window **(A)**; 2) Making the left-open single-flap **(B)**; 3) Fixing the esophagus and opening a gastric mucosal window **(C)**; 4) Anastomosing by linear staplers **(D)**; 5) Closing of the common opening with barbed wire **(E)**; 6) Covering the left-open single-flap and suturing with barbed wires **(F)** (20).

As shown in **Figure 2**, patients in the trial group will receive the left-open single-flap technique after proximal gastrectomy, which involves the following (20):

1) Marking an “⊐”-shaped mucosal window (A): The mucosal window is located on the anterior wall of the stomach near the lesser curvature, 3-4 cm from the cut edge, and a sideways “⊐” shape is marked measuring (2.5-3.5) x 3.5 cm area with methylene blue.
2) Making a left-open single flap (B): an electric knife is used to peel off the plasma membrane and muscle layers, paying attention to the protection of blood vessels and avoiding rupture of the mucosal window.
3) Fixing the esophagus and opening a gastric mucosal window (C): the posterior wall of the esophagus, approximately 5 cm from the severed end, is fixed with four sutures to the top of the anterior wall of the remnant stomach. The gastric mucosal window (upper and lower edges of the anastomosis) is made by opening the mucosal layer under the single muscle flap at the lower left side of the flap. The length of the window is determined by the caliber of the esophagus.
4) Anastomosing by linear staplers (D): the left posterior wall of the esophagus and anterior wall of the gastric mucosal window are anastomosed by linear staplers with an insertion length of 2.5-3 cm.
5) Closing of the common opening (E): the common opening is closed with a full layer of continuous sutures using a barbed wire.
6) Covering the left-open single flap and suturing with barbed wires (F): the anastomosis is covered by a left-open single flap, and the edge of the flap is continuously sutured to the anterior gastric wall with barbed wires. A “⊐”-shaped structure is formed.

**Figure 3:**
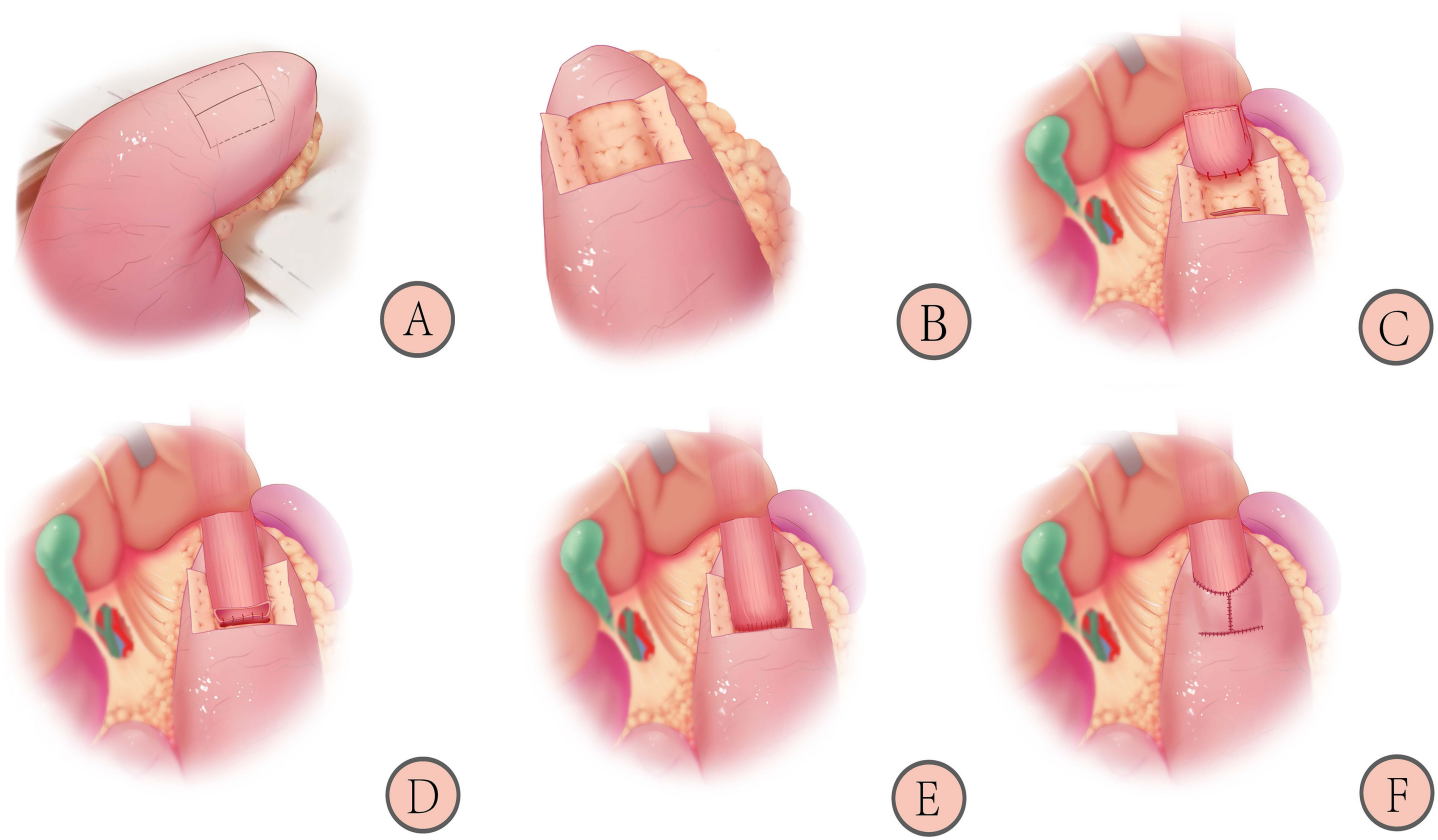
Schematic diagram of reconstruction after proximal gastrectomy with the double-flap technique: 1) Marking the “H”-shaped mucosal window **(A)**; 2) Making the double-flap **(B)**; 3) Fixing the esophagus and opening the gastric mucosal window **(C)**; 4) Completing continuous hand suture **(D, E)**; 5) Covering the double-flap and suturing with barbed wires **(F)** (21).

As shown in **Figure 3**, patients in the control group will receive the double-flap technique after proximal gastrectomy, which includes the following (21):

1) Marking the “H”-shaped mucosal window (A): the mucosal window is located on the anterior wall of the remnant stomach near the lesser curvature, and the “H”-shaped (2.5~3.5) cm×3.5 cm area is marked with methylene blue.
2) Make a double flap (B): an electric knife is used to peel off the plasma membrane layer and muscle layer, paying attention to the protection of blood vessels and avoiding rupture of the mucosal window.
3) Fixing the esophagus and opening the gastric mucosal window (C): the posterior wall of the esophagus, approximately 5 cm from the severed end, is sutured to the top of the anterior wall of the remnant stomach with 3-4 stitches to keep the anastomosis flat and prevent reflux. The gastric mucosal window is made by opening the mucosal layer below the double flap, and the width is similar to that of the esophagus.
4) Completing continuous hand suture (D and E): the entire esophageal wall is sutured to the gastric mucosa using a barbed wire with a complete continuous inversion. The point of entry is the left side of the upper edge of the gastric mucosa. The direction of entry is entering the gastric mucosal layer and penetrating from the esophageal plasma membrane layer. The direction of the suture is from left to right until the left side of the gastric mucosa upper edge. The direction of the suture at the lower edge is the opposite.
5) Covering the double flap and suturing with barbed wires (F): the anastomosis is covered by a double flap, and the lower edge of the flap is continuously sutured to the anterior gastric wall with barbed wires. The double flap is obliquely reinforced to the esophageal epithelium without mutual sutures, and a “Y”-shaped collar-like structure is formed.

## Clinical data collection

According to the privacy policy, only the researcher will know the patient’s identity and various information. Clinical information will be recorded by the investigators in the case report form and on the web platform (http://www.medresman.org.cn). The patients’ clinical data will include general information, previous medical history, previous surgical history, laboratory findings (preoperative and postoperative blood tests, biochemical indicators, and tumor markers), upper gastrointestinal endoscopy, imaging findings, the incidence of postoperative reflux esophagitis, the incidence of anastomotic leakage, the incidence of anastomotic stricture, operative time and intraoperative blood loss. The schedule of data collection in this study is shown in **Table 3**.

**Table 3:** The 6 medical institutions participating in the clinical trial.

Assessment time point	Preoperation (14-1 days)			Intraoperation	Postoperation							
	Enrollment	Allocation	Baseline		1-14 days	1st month	3rd month	6th month	12th month	18th month	24th month	36th month
Inclusion and exclusion	√											
Written informed consent	√											
Patients allocation		√										
Basic data collection			√									
Preoperative management			√									
Operation information				√								
Frozen-section examination				√								
Questioning			√		√	√	√	√	√	√	√	√
Physical examination			√		√	√	√	√	√	√	√	√
Blood examination items			√		√	√	√	√	√	√	√	√
Gastrointestinal endoscopy			√									
Esophagogram fluoroscopy					√	√	√	√	√	√	√	√
Imaging items			√		√	√	√	√	√	√	√	√
Other assessment tools			√	√	√	√	√	√	√	√	√	√

√Indicates the need to collect clinical data.

## Collection, preservation, and management of biochemical specimens

In this study, blood samples will be collected from subjects to monitor blood biochemical indicators and tumor markers. After testing, all samples will be destroyed in strict accordance with laboratory regulations.

## Sample size estimate and statistical analysis

The left-open single-flap technique after proximal gastrectomy is a new and improved procedure in our center, and national or international studies comparing clinical outcomes after reconstruction with the double-flap technique have not been conducted. Kuroda S et al. (22) showed that the incidence of reflux esophagitis, performed by endoscopy at 1.0 years (median) after the double flap technique, was 10.6% for all grades. This finding was considered more in line with “real‐world data”.

According to the database of this study, we designed a noninferiority study with a noninferiority margin of 10% (α=0.05, β=0.20, δ =0.10, 80% power, 10% dropout rate). The test statistic used is the one-sided Z test (unpooled) by PASS 15.0.5 software.

The result is N = 250. Therefore, 125 patients will be enrolled in each of the 2 groups.

## Study endpoints

The postoperative complications are classified according to the Clavien–Dindo grading system.

### Primary study endpoints

1) Incidence of reflux esophagitis (22);

### Secondary study endpoints

1) Incidence of anastomotic stricture;
2) Overall postoperative complication rate;
3) Incidence of anastomotic fistula;
4) Operation time;
5) Blood loss volume;
6) Postoperative mortality rate;
7) R0 resection rate;
8) Overall resection rate;
9) Intraoperative complication rate;
10) Postoperative severe morbidity rate;
11) Postoperative recovery course;
12) 3-year overall survival rate;
13) 3-year disease-free survival rate;
14) Recurrence pattern;
15) The length of ICU stay;
16) Lengths of admission;
17) Length of post operational stays;
18) Nutritional status.

## Follow-up

The start time of this study was August 1, 2022. The preliminary completion time is July 31, 2024. Follow-up will be planned for three years, and the study completion time will be July 31, 2027.

Esophagogram fluoroscopy will be performed 6 days after surgery to evaluate anastomotic complications. Subsequent follow-ups will be performed at 1, 3, 6, 12, 24 and 36 months. The follow-up will include questioning, physical examination, gastrointestinal endoscopy, blood examination items (peripheral blood routine, serum iron, vitamin B12, folic acid, blood biochemistry and serum tumor markers), and imaging items (chest imaging, esophagogram fluoroscopy, and whole abdomen enhanced CT). All examinations will be recorded to evaluate the presence of tumor recurrence or metastasis, the survival status of patients and the occurrence of complications. Other assessment tools will be used according to the specific situation, such as color ultrasound of other sites, whole-body bone scan, PET-CT, etc. The follow-up schedule in this study is shown in **Table 3**.

The diagnosis of postoperative reflux symptoms is based on a combination of symptom presentation, endoscopic evaluation of esophageal mucosa, reflux monitoring, and response to therapeutic intervention (23–25). Heartburn and regurgitation remain the typical symptoms of postoperative reflux symptoms. The control steps of postoperative reflux symptoms are shown in the **Table 4**.

**Table 4:** The control steps of postoperative reflux symptoms.

Management	Nonpharmacologic lifestyle modifications	Weight managemen	1. Weight loss in overweight and obese patients for improvement of postoperative reflux symptoms. 2. For the patients with regurgitation or belch predominant symptoms; but we do not recommend baclofen in the absence of objective evidence of postoperative reflux symptoms.
		Body positioning	1. Elevating the head of the bed. 2. Staying upright during and after meals.
		Diet modification	1. Avoidance of “trigger foods”. 2. Avoiding meals within 2–3 hours of bedtime. 3. Tobacco and alcohol cessation. 4. Avoidance of late night meals and bedtime snacks.
	Pharmacologic therapy	Proton pump inhibitors	1. Patients presenting with troublesome heartburn, regurgitation, and/or non-cardiac chest pain without alarm symptoms a 4- to 8-week trial of single-dose PPI therapy. 2. With inadequate response, dosing can be increased to twice a day or switched to a more effective acid suppressive agent once a day. When there is adequate response, PPI should be tapered to the lowest effective dose. 3. If the response to one type of PPI is not sufficient, switching to another type of standard-dose PPIs may be considered (administration 30–60 minutes before a meal and on-demand therapy/ maintenance therapy). 4. For patients with postoperative reflux symptoms who do not have erosive esophagitis or Barrett’s esophagus, and whose symptoms have resolved with PPI therapy, an attempt should be made to discontinue PPIs. 5. We recommend maintenance PPI therapy indefinitely or antireflux surgery for patients with Los Angeles grade C or D esophagitis. 6. Endoscopic evaluation:Objective testing with upper GI endoscopy is warranted in PPI non-response, presence of alarm signs/symptoms, isolated extra-esophageal symptoms, or in patients who meet criteria to undergo screening for Barrett’s esophagus. In the absence of confirmed erosive disease or Barrett’s esophagus on endoscopy, prolonged wireless pH monitoring off PPI therapy is utilized to assess esophageal acid exposure.
		Potassium-competitive acid blockers	The efficacy is comparable to proton pump inhibitors for 4 weeks and 8 weeks, which are recommended as an initial treatment of postoperative reflux symptoms.
		H2 receptor antagonists	Patients with nocturnal symptoms.
		Alginate antacids	Patients with breakthrough symptoms.
		Baclofen	Patients with regurgitation or belch predominant symptoms; Don't recommend in the absence of objective evidence of postoperative reflux symptoms.
		Prokinetics	Patients with coexistent gastroparesis.
		Behavioral therapist	Patients with functional heartburn or reflux disease associated with esophageal hypervigilance, reflux hypersensitivity, and/or behavioral disorders (hypnotherapy, cognitive behavioral therapy, diaphragmatic breathing, and relaxation strategies).
	Surgery		Patients with Los Angeles grade C or D esophagitis.

In this study, the patients should be reexamined at the hospital where the surgery was performed, but cases of outside hospital examination will not be excluded (outside hospital reexamination should be conducted at a tertiary care hospital). A follow-up specialist will follow up and record the results of each examination.

All patients refuse to be followed up according to the above protocol will be recorded as lost cases and analyzed together with cases meeting the study criteria at the end of the study.

## Written informed consent forms

The informed consent process was approved by the internal review board/independent ethics committee. If any changes occur during the study, they will be resubmitted for review.

Informed consent procedures will be implemented in strict compliance with relevant Chinese laws. Original informed consent will be retained in writing by the investigator.

We will rigorously protect patient privacy: The collection, transmission, process and storage of participant data will comply with data security and privacy protection regulations.

## Monitoring of the study

The study protocol, which was submitted to the ethical review committee of the health administration department, is in line with the relevant regulations in China and the Measures for Ethical Review of Biomedical Research Involving Human Beings (2007).

We will retain video recordings and unedited image files of all patients throughout the surgery. The organizers will review and monitor the quality of the surgery. The main objectives will be performed for these purposes: 1) to confirm the rationality of the surgical approach, the extent of lymph node dissection and the minimally invasive nature of the incision; 2) to verify the original data of all subjects to confirm consistency with the CRF; and 3) to regularly assess the progress of the study at each center to ensure that it was carried out according to the plan.

## Patient and public involvement

Patients and the public will not be involved in our process of design, recruitment, clinical treatment, measurement of outcomes and analysis of the data.

## Discussion

For patients diagnosed with Siewert III esophagogastric junction adenocarcinoma and early-stage upper gastric cancer, current clinical guidelines recommend proximal gastrectomy (17, 18). Compared with total gastrectomy, proximal gastrectomy can maintain part of the storage and digestive function of the stomach, which has greater advantages for nutrient absorption and weight maintenance (5–8). Therefore, this approach is more commonly used in East Asia.

Unavoidably, patients’ postoperative quality of life is severely affected by postoperative complications (7). Wang S et al. (8) found that proximal gastrectomy is also associated with many complications, most notably reflux esophagitis, which causes heartburn, chest pain, acid reflux, and anorexia. These postoperative complications can severely reduce the quality of life after surgery.

Consequently, the choice of a reasonable approach to reconstruct the digestive tract after proximal gastrectomy and addressing complications, such as reflux esophagitis, remain a challenge for clinicians (10–13). Therefore, an increasing number of clinical studies have focused on improving reconstruction modalities after proximal gastrectomy, such as esophagogastrotomy (EG), jejunostomy (JI), jejunal pouch placement (JPI) and double tract reconstruction (DTR).

Among various gastrointestinal reconstruction methods, the double-flap technique is clinically effective, wrapping around and increasing the pressure of anastomosis through a unidirectional flap. This structure is similar to the “reconstructed cardia” structure, which can improve the antireflux effect. Saze Z et al. (16) found that the double-flap technique did not result in postoperative reflux esophagitis and had the smallest postoperative weight change ratio and the lowest prevalence of gastric residual at 12 months after surgery compared with direct esophagogastrostomy, jejunostomy and double tract reconstruction.

However, double-flap technique is also associated with shortcomings, such as a complicated surgical suture technique, difficult operation, strict surgical indications and high incidence of postoperative anastomotic stenosis (26–28).

To address the shortcomings of the above procedure, we established a multicenter, prospective, randomized controlled trial to modify the flap-making procedure for the double-flap technique: a left-open single flap will be used instead of a double flap to cover the anastomosis, acting as a “sphincter” and providing a tunneling effect. Preliminary data from our center showed that patients have excellent postoperative results. We will explore and summarize our initial clinical experience and apply it to the development of the treatment protocol. If this study meets the expected results of the trial protocol, it will bridge the gap between the complicated operative procedure and longer operative time of the double-flap reconstruction style and further improve the postoperative quality of life of patients. These outcomes will be landmark improvements in patient prognosis and provide additional high-level research evidence for the standardization of gastrointestinal reconstruction protocols after proximal gastrectomy.Strengths and limitations of this study

Strengths: This was a prospective, large sample, multicenter randomized controlled trial that systematically compared the efficacy of 2 methods of gastrointestinal reconstruction after proximal gastrectomy. In previous studies, the left-open single-flap technique had not been reported.

Limitations: Japanese guidelines recommended that the common methods of gastrointestinal reconstruction after proximal gastrectomy include esophagogastrotomy (EG), jejunostomy placement (JI), jejunal pouch placement (JPI) and double-tract reconstruction (DTR). Nevertheless, we compared only 2 of these reconstruction methods in our study.

## Consent form for data publication

All authors agree to publish and all participants will sign a data release consent form.

## Data availability statement

The original contributions presented in the study are included in the article/supplementary material. Further inquiries can be directed to the corresponding authors.

## Ethics statement

The studies involving human participants were reviewed and approved by Chinese Ethics Committee of Registering Clinical Trials. The patients/participants provided their written informed consent to participate in this study.

## Author contributions

Concept Proposal: XL; Survey and Data Summary: QY, WW, RG, CY, JY, and DD; Data Collection, analysis and statistics: QY, WW, ZM, CY, HZ, and JW; Specific scheme implementation: GJ, XL, QY, WW, ZM, CY, RG, DD, HZ, JW, JL, and XY; Research Regulatory: GJ and XL; Writing – Draft: QY and WW; Writing-Proofreading and Editing: XL and QY. All authors approved the final version of this manuscript.

## Funding

This study was funded in part by grants from the Scientific and Technological Innovation Team Plan of Shaanxi Province (2021TD-43) by GJ.

## Acknowledgments

The authors thank all the medical staff and patients who contribute to this study.

## Conflict of interest

The authors declare that the research was conducted in the absence of any commercial or financial relationships that could be construed as a potential conflict of interest.

## Publisher’s note

All claims expressed in this article are solely those of the authors and do not necessarily represent those of their affiliated organizations, or those of the publisher, the editors and the reviewers. Any product that may be evaluated in this article, or claim that may be made by its manufacturer, is not guaranteed or endorsed by the publisher.

## Abbreviations

GC, gastric cancer; RCT, randomized controlled study; EGC, early gastric cancer; CRF, Case Report Form; EG, esophagogastrotomy; DFT, double-flap technique; LOSF, left-open single-flap.
